# Development of the blood–brain barrier within the paraventricular nucleus of the hypothalamus: influence of fetal glucocorticoid excess

**DOI:** 10.1007/s00429-014-0787-8

**Published:** 2014-05-11

**Authors:** Krystle A. Frahm, Stuart A. Tobet

**Affiliations:** 1 Program in Cell and Molecular Biology, Colorado State University, 1617 Campus Delivery, Fort Collins, CO 80523-1617 USA; 2 Department of Biomedical Sciences, Colorado State University, Fort Collins, CO USA; 3 School of Biomedical Engineering, Colorado State University, Fort Collins, CO USA

**Keywords:** Blood–brain barrier, Prenatal stress, Pericytes, Paraventricular nucleus of the hypothalamus

## Abstract

The blood–brain barrier (BBB) is a critical contributor to brain function. To understand its development and potential function in different brain regions, the postnatal (P) BBB was investigated in the mouse cortex (CTX), lateral hypothalamus, and paraventricular nucleus of the hypothalamus (PVN). Brains were examined on postnatal days (P)12, P22 and P52 for BBB competency and for pericytes as key cellular components of the BBB demarcated by immunoreactive desmin. Glucocorticoid influences (excess dexamethasone; dex) during prenatal development were also assessed for their impact on the blood vessels within these regions postnatally. At P12, there was significantly more extravascular leakage of a low molecular weight dye (fluorescein isothiocyanate) in the CTX than within hypothalamic regions. For pericytes, there were low levels of desmin immunoreactivity at P12 that increased with age for all regions. There was more desmin immunoreactivity present in the PVN at each age examined. Fetal dex exposure resulted in decreased blood vessel density within the PVN at P20. In the CTX, dex exposure increased BBB competency, in contrast to the PVN where there was a decrease in BBB competency and increased pericyte presence. Overall, unique alterations in the functioning of the BBB within the PVN may provide a novel mechanism for fetal antecedent programming that may influence adult disorders.

**Electronic supplementary material:**

The online version of this article (doi:10.1007/s00429-014-0787-8) contains supplementary material, which is available to authorized users.

## Introduction

The vasculature of the brain differs from the periphery in several characteristics. A key difference is the blood–brain barrier (BBB), which restricts access to the brain parenchyma through a complex network of tight junction proteins, proteoglycans, endothelial cells, basal lamina, vascular smooth muscle cells, pericytes and glial cells (Norsted et al. 2008). As more research implicates the BBB in disease onset and progression (Gosselet et al. 2011; Daneman 2012; Abbott and Friedman 2012), its development and function becomes a more important area of focus.

Pericytes play a role in the development and integrity of the BBB. Immunoreactive desmin provides a reliable biochemical marker of pericytes (Hellstrom et al. 1999). When pericytes are deficient (e.g., PDGF KO mice; Armulik et al. 2010), there is an improper astrocyte end-feet distribution and an increase in injected tracers present in brain parenchyma. Neuronal degeneration resulting in memory impairment is preceded by pericyte loss (Bell et al. 2010). During disease states such as stroke, pericytes can migrate into coordinate blood flow regulation, permeability of the BBB, and reestablishment of neurovascular units (Liu et al. 2012).

Although much BBB research focuses on the cerebral cortex (CTX), there is no a priori reason to assume that all other brain regions maintain the BBB under the same rules. For example, circumventricular organs maintain a more permeable BBB within the brain but vary in their permeability (Morita and Miyata 2012). The current study focused on the paraventricular nucleus of the hypothalamus (PVN) that contains a 3–5 fold denser matrix of blood vessels than surrounding brain regions (Finley 1938; Ambach and Palkovits 1974; van den Pol 1982) and may play by different rules. The PVN houses neurons containing corticotropin-releasing hormone (CRH), arginine vasopressin and angiotensin that control physiological homeostasis, vasomotor tone, and stress responses (Tobet et al. 2013). The vascular density arises postnatally and varies from rostral to caudal (Frahm et al. 2012). The greater density in the rostral and mid region corresponds with the general location of neuroendocrine neurons (Biag et al. 2012). Altering exposure of specific neurons to peripheral signals through a compromised BBB may contribute to various diseases and disorders (Quaegebeur et al. 2011). Within the PVN, decreases in BBB integrity might have effects amplified by the threefold greater vascular network (Goldstein et al. 2013).

Prenatal glucocorticoid excess leads to long-term functional consequences in adulthood (reviewed in Harris and Seckl 2011; Tobet et al. 2013). At a cellular level, prenatal glucocorticoids alter glucocorticoid receptor expression in the hippocampus in adulthood (Levitt et al. 1996) and increase CRH levels within the PVN (Welberg et al. 2001). Concerning the vasculature, prenatal glucocorticoid excess may decrease blood vessel density (Neigh et al. 2010; Vinukonda et al. 2010) and increase pericyte coverage (Vinukonda et al. 2010). A goal of the current study was to assess whether the dense blood vessel network in the PVN is impacted by fetal glucocorticoid excess.

The current study characterized the postnatal development of the BBB and desmin-immunoreactive pericytes in the CTX, lateral hypothalamus (LH) and PVN. Fetal exposure to dex resulted in enhanced BBB integrity in the CTX, while the same treatment resulted in decreased blood vessel density and BBB integrity within the PVN. The divergence of effect may be related to a selective increase in desmin-immunoreactive pericyte coverage in the PVN in offspring exposed to dex during pregnancy.

## Materials and methods

### Animals

For experiments selectively examining BBB development, the mice used were from a mixed C57BL6/S129/CBA background (Solomon et al. 2012) and for experiments examining the influence of prenatal dex mice were from an FVB/N background. Males and females combined by genotype after analysis (ANOVA sex × treatment × region at P20 *p* > 0.50) indicated no significant differences by sex. Mice were mated overnight and the day of a visible plug was designated as embryonic day 0 (E0). Pregnant dams were injected with either the synthetic glucocorticoid dexamethasone (0.1 mg/kg, Sigma, Inc.; Hadoke et al. 2006; O’Regan et al. 2004) or vehicle once daily from E11-17. The day of birth was designated P0. For tissue collection, mice were anesthetized using ketamine (80 mg/kg) and xylazine (8 mg/kg) and transcardially perfused with heparinized PBS (pH 7.4) containing fluorescein isothiocyanate (FITC, Thermoscientific, MW 389.4) followed by 4 % paraformaldehyde in 0.1 M phosphate buffer (pH 7.4; modified from Miyata and Morita 2011). To examine blood vessel density, a separate subset of mice was anesthetized by inhaling isoflurane (Vet One) and brains were removed and immersion fixed with 20 ml 4 % paraformaldehyde in 0.1 M phosphate buffer. For all mice, brains were removed, post fixed overnight, then changed into 0.1 M phosphate buffer for storage at 4 °C. Body weights were measured and sex determination was made through direct inspection of the gonads. There were at least three separate litters combined for analysis of each treatment.

Mice were maintained in plastic cages with aspen bedding (autoclaved Sani-chips, Harlan Teklad, Madison, WI, USA) in the Painter Building of Laboratory Animal Resources at Colorado State University. Food (#8640, Harlan Teklad, Madison, WI, USA) with filtered tap water and environmental enrichment provided ad libitum in a 14/10 h light/dark cycle. Animal care and handling was in accordance with the Colorado State University Animal Care and Use Committee guidelines.

### Immunohistochemistry

Tissue was processed as previously described (Frahm et al. 2012, 2013). In brief, brains were embedded in 5 % agarose and cut coronally into 50 μm thick sections using a vibrating microtome (Leica VT1000S). Free-floating serial sections were collected in 0.05 M phosphate-buffered saline (PBS, pH 7.4). Excess unreacted aldehydes were neutralized in 0.1 M glycine for 30 min followed by 0.5 % sodium borohydride for 15 min. Sections were washed in PBS then incubated in a blocking solution (5 % normal goat serum (NGS), 0.5 % Triton X-100 (Tx), and 1 % hydrogen peroxide in PBS) for at least 30 min. Sections were then incubated in primary antiserum directed against platelet endothelial cell adhesion molecule (PECAM also known as CD31, 1:30; BD Biosciences, San Jose, CA) or desmin (1:200; DAKO) in 1 % BSA and 0.5 % Tx. Antisera for other pericyte markers were tested and found to label additional cell types. Therefore, desmin was used for all experiments because it reliably and selectively labeled pericytes. For desmin, sections were processed for antigen retrieval (Dellovade et al. 2001). In place of the standard processing steps prior to antisera application detailed above, sections were washed in room temperature PBS for 15 min followed by a 1 h wash in sodium citrate (0.05 M, pH 8.6). The sections were then placed into sodium citrate buffer preheated to 80 °C for 30 min. They were then allowed to slowly come back to room temperature (~30–35 min) after which they were returned to PBS for an additional 15 min of washes. All sections were incubated for two nights at 4 °C in primary antisera. Sections were then washed in room temperature with 1 % NGS and 0.02 % Tx in PBS. Sections were incubated with the appropriate secondary antibodies for 2 h for either biotin conjugated donkey anti-rat antiserum (1:1,000; Jackson Immunoresearch, West Grove, PA), Cy3 conjugated anti-rabbit (1:200; Jackson Immunoresearch) or Cy3 conjugated anti-mouse (1:200; Jackson Immunoresearch) in PBS containing 1 % NGS and 0.32 % Tx. For PECAM, sections were incubated in a Vectastain reagent (3 µl/ml solutions A and B––Vectastain ABC Elite kit; Vector Laboratories, Burlingame, CA) at room temperature for 1 h. After 1 h of washing in Tris-buffered saline (pH 7.5), reaction product was developed over 5 min in Tris-buffered saline containing 0.025 % diaminobenzidine, 0.02 % nickel, and 0.02 % hydrogen peroxide.

### Analysis

For blood vessel density, images were acquired for the PVN, LH and CTX using an Olympus BH2 microscope with an Insight QE digital camera in Spot Advanced Software. The section with the densest vascular network was selected by an investigator blind to treatment group for each PVN region (rostral, mid, caudal) for analysis (Frahm et al. 2012). Image representation for the regions selected for analysis (CTX, PVN, and LH) is provided in Supplemental Fig. 1. Total number of blood vessel branches and length were used to characterize the density in each region of interest containing the PVN. For blood vessel length, images were light corrected (Image J, version 1.43u) then analyzed for length using angiogenesis tube formation (Metamorph Software, version 7.7.0.0, Molecular Devices, Inc.). Branch points were manually identified and counted using Image J (cell counter). Blood vessel width was quantified by dividing total area by total length. For desmin and FITC, images were acquired on a Zeiss 510-Meta laser-scanning confocal microscope. FITC was imaged using a 488/543 nm bandpass filter and emission detected using a 505/530 nm bandpass emission filter. Cy3 for desmin was imaged using a 488/543 nm bandpass filter and emission detected using a 585/615 nm bandpass emission filter. Z-stacks were taken with six optical sections taken every 3 μm obtained at 40× magnification using an oil immersion objective. FITC does not remain in blood vessels but rather accumulates in endothelial cell nuclei (Miyata and Morita 2011). Therefore, to view the vascular network within the brain, we compiled Z-stacks for analysis. Extravascular leakage was analyzed using open-source CellProfiler (available from the Broad Institute at www.cellprofiler.org). Blood vessels were identified and a 10-pixel expansion was mapped from each blood vessel to create a mask to quantify leakage. This intensity was divided by FITC intensity within blood vessels to account for differences in perfusions. A representation of the CellProfiler analysis is provided in Supplemental Fig. 2. Because blood vessel density varies, final values were normalized to blood vessel area within the same section. For desmin analysis, sections were measured for area of immunoreactive and additionally were normalized to blood vessel area using Metamorph software. Representative images for figures were normalized for optimal contrast in Adobe Photoshop (version CS for Macintosh). Statistical significance was determined by two-way ANOVAs: age × region for developmental studies and treatment × region for dex studies using SPSS software (version 21 for Macintosh, SPSS Inc., Chicago, IL). In all cases, region was considered as a repeated measure. This was followed by post hoc comparisons based on Bonferroni correction. Values of *p* < 0.05 were considered statistically significant and are reported as mean ± SEM.

**Fig. 1:**
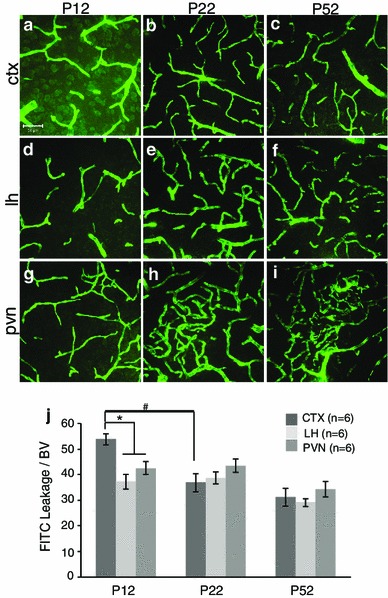
Postnatal blood–brain barrier development in the mouse cortex (CTX), lateral hypothalamus (LH) and paraventricular nucleus of the hypothalamus (PVN) at P12, P22 and P52. Example confocal images for each region are provided in panels **a**–**i**, and a quantitative summary by graph in **j**. There was a significant increase in extravascular FITC leakage in the CTX (**a**) compared to the LH (**d**) and PVN (**g**) at P12 (**j**; *p* < 0.05). Between P12 and P22 there was a significant decrease in extravascular FITC leakage specifically in the CTX (**a**, **b**; *p* < 0.05). At P22, there were no significant differences observed in extravascular FITC leakage between brain regions (**b**, **e**, **h**). At P52, there were no significant differences in extravascular FITC leakage (**c**, **f**, **i**) compared to P22 or between brain regions (**j**). Number of animals per group (*n* = 6) is provided in the code for the *bars* panel **j**. Significant differences between regions indicated by *asterisk* and for age as *hash*. *Scale bar* 50 µm in panel **a**, which applies to all images

**Fig. 2:**
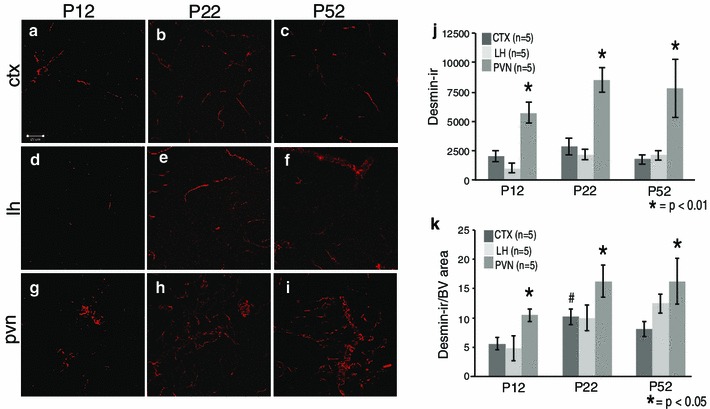
Postnatal desmin-immunopositive pericyte coverage in the mouse cortex (CTX), lateral hypothalamus (LH) and paraventricular nucleus of the hypothalamus (PVN) at P12, P22 and P52. Example confocal images for each region are provided in panels **a**–**i**, and a quantitative summary by graph in **j** and **k**. There was a significant increase in desmin-immunoreactive pericyte coverage in the PVN (**g**) compared to the CTX (**a**) and LH (**d**) at P12 (**j**, **k**; *p* < 0.05). At P22, there was a significant increase in desmin-immunoreactive pericyte coverage in the CTX (**b**) and PVN (**h**) compared to P12 (**j**, **k**; *p* < 0.05). There were no significant differences in any brain region between P22 and P52 for desmin-immunoreactive pericyte coverage (**j**, **k**). There was an overall significant increase in desmin-positive pericyte coverage for the PVN at all ages (**g**–**i**) compared to the LH (**d**–**f**) and CTX (**a**–**c**) for all ages (**j**, **k**; *p* < 0.05). Number of animals per group (*n* = 5) is provided in the code for the *bars* panels **j** and **k**. Significant differences between regions indicated by *asterisk* and for age as *hash*. *Scale bar* 50 µm in panel **a**, which applies to all images

## Results

### Age- and region-dependent changes in BBB competency

The current study found changes in vasculature structure and extravascular leakage within the CTX, LH and PVN from P12 to P22 and P52. These time points were chosen based on the significant increase in PVN angiogenesis over these ages (Frahm et al. 2012). On P12, the BBB in the CTX was less competent compared to the LH and PVN. There was significant extravascular FITC leakage within the CTX at P12 compared to the LH and PVN (Fig. 1a, d, g, j; *p* < 0.05). This high level of extravascular FITC was not observed in the hypothalamic regions of LH and PVN at P12. At P22, there was significantly less extravascular FITC leakage in the CTX compared to P12 (Fig. 1b, j; *p* < 0.05). There were no significant differences between brain regions concerning extravascular FITC leakage at P22 (Fig. 1b, e, h). At P52, the BBB appeared fully functional as extravascular FITC leakage did not change in CTX, LH, and PVN (Fig. 1c, f, i, j) compared to the same brain regions at P22 (Fig. 1b, e, h, j). These findings suggest that the BBB develops at different rates in the CTX compared to the hypothalamic brain regions examined.

### Changes in desmin immunoreactive pericytes by age and region

**Fig. 3:**
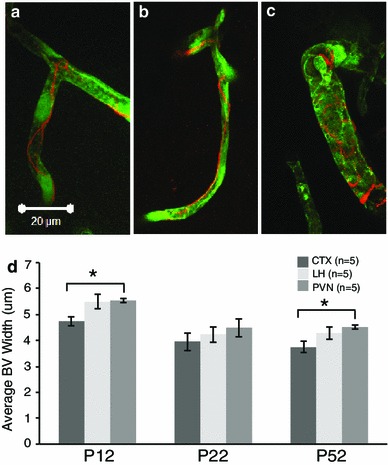
Blood vessels in the paraventricular nucleus of the hypothalamus (PVN) were wider than in the mouse cortex (CTX) at P12 and P52. Higher magnification of blood vessels at P52 visualized with fluorescein isothiocyanate perfusion in the CTX, lateral hypothalamus (LH) and PVN show that desmin morphology varied between brain regions with the PVN (**c**) having more of a wrapping pattern compared to the CTX (**a**) and LH (**b**). The wrapping may be related to a significantly greater blood vessel width in the PVN compared to the CTX at P12 and P22 (**d**, *p* < 0.05). There were no significant differences at P22 or in the LH when compared with the CTX or PVN at any age. Number of animals per group (*n* = 5) is provided in the code for the bars panels **j** and **k**. Significant differences between regions indicated by *asterisk*. *Scale bar* 20 µm in panel **a**, which applies to all images

Concerning postnatal and region-specific pericyte development, results showed significantly greater desmin-immunoreactive pericyte coverage at P22 and P52 compared to P12 (Fig. 2a–i; *p* < 0.01). For different brain regions, there was significantly more desmin-immunoreactive pericyte coverage at P12 in the PVN (Fig. 2g–i) compared to the LH (Fig. 2d–f) and CTX (Fig. 2a–c). For the CTX, there was a significant increase in desmin-immunoreactive pericyte coverage between P12 and P22 (Fig. 2a, b, j, k). At all ages examined, the PVN had significantly more desmin-immunoreactive pericyte coverage than the LH and the CTX (Fig. 2j; *p* < 0.01). When blood vessel density was taken into account, the PVN still had significantly more desmin-immunoreactive pericyte coverage than the CTX (Fig. 2k; *p* < 0.05). At P52, this increase in desmin-immunoreactive pericyte coverage was due to the morphology of the pericytes in the PVN (Fig. 3c) compared to the CTX (Fig. 3a). Desmin in the adult mouse labels processes running along small diameter and encircling larger diameter capillaries (Hellstrom et al. 1999). The pattern of desmin-immunoreactive pericyte coverage in the PVN showed a wrapping pattern around blood vessels while in the CTX more often it extended along the blood vessels. There were no differences in desmin-immunoreactive pericyte coverage in the LH (Fig. 3b) compared to the CTX or PVN after 50 days of age. To determine if the difference in pericyte coverage coincided with the size of blood vessels, blood vessel width was quantified (Fig. 3). Blood vessel widths were greater in the hypothalamus (LH––Fig. 3b, PVN––Fig. 3c) compared to the CTX (Fig. 3a). Quantification showed a statistically significant greater blood vessel width in the PVN (but not the LH) compared to the CTX (Fig. 3d; *p* < 0.05) indicating that at P52, the greater desmin-immunoreactive pericyte coverage in the PVN (Fig. 2i–k) was associated with an increase in blood vessel width (Fig. 3d). To examine if this was due to the presence of larger arterioles, antibodies against smooth muscle actin (SMA), a marker for smooth muscle cells that surround cerebral arteries or arterioles (Ladecola 2004) was examined. SMA immunoreactivity was observed in the brain, however, not within the PVN (data not shown) suggesting the larger width of blood vessels within the PVN was not due to the presence of arterioles, although this did not rule out the presence of venules. In general, desmin-positive pericyte coverage increased postnatally, varied between brain regions, and was related to blood vessel width.

### Fetal dex exposure led to altered vascular characteristics at P20

**Fig. 4:**
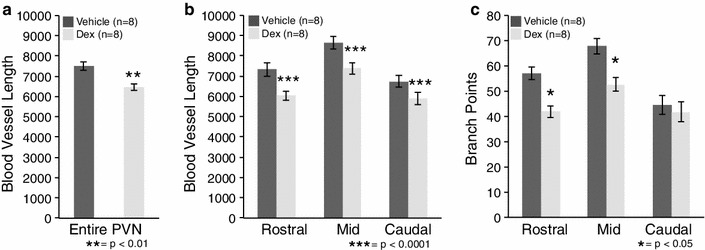
Prenatal exposure to dexamethasone (dex) impacted blood vessel density in the postnatal mouse paraventricular nucleus of the hypothalamus (PVN) at P20. There was a significant decrease in blood vessel length for the entire PVN for dex-treated compared to vehicle-treated mice (**a**, **p < 0.01). There was also a region-specific significant decrease in blood vessel length in the rostral, mid and caudal regions of the PVN in dex-treated compared to vehicle-treated mice (**b**, ***p < 0.0001). For branch points, there was only a significant decrease in the rostral and mid PVN in dex-treated compared to vehicle-treated mice (**c**, *p < 0.05). Number of animals per group (*n* = 8) is provided in the code for the *bars* in each panel

Blood vessels that are potentially newly formed and not yet fully functional are not identified by vascular perfusion with FITC (Frahm et al. 2013). Therefore, immunoreactive PECAM was utilized to visualize the more complete endothelial cell population. PECAM revealed an overall 13 % decrease in blood vessel length in the PVN for dex-treated compared to vehicle-treated mice at P20 (Fig. 4a; *p* < 0.01). Offspring of dex-treated mothers had significantly less total blood vessel length across all regions of the PVN (Fig. 4b; *p* < 0.01), while decreased branch points were restricted to the rostral and mid regions compared to vehicle-treated (Fig. 4c; *p* < 0.05). Brains perfused with FITC were also examined and dex-exposed offspring had less blood vessel density compared to vehicle-treated (data not shown). There were no significant differences in blood vessel length or branch points in the LH or CTX due to dex-treatment (data not shown). This indicates that prenatal exposure to dex impacts blood vessels within the PVN of young offspring.

### Fetal dex exposure led to altered BBB competency at P20

**Fig. 5:**
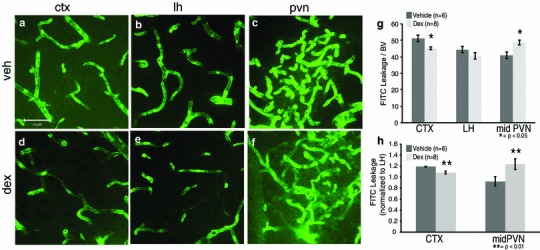
Prenatal exposure to dexamethasone (dex) impacted blood–brain barrier development in the mouse cortex (CTX) and paraventricular nucleus of the hypothalamus (PVN) at P20. Example confocal images for each region are provided in panels **a**–**f**, and a quantitative summary by graph in **g** and **h**. In the CTX, there was a significant decrease in extravascular FITC leakage in dex-treated compared to vehicle-treated mice (**a**, **d**, **g**; *p* < 0.05). For the PVN, there was a significant increase in extravascular FITC leakage in offspring of dex-treated compared to vehicle-treated mice in the mid region (**c**, **f**, **g**; *p* < 0.05). There was no impact of fetal dex observed in the lateral hypothalamus (LH; **b**, **e**, **g**). Number of animals per group is provided in the code for the *bars* in panels **g** and **h**. Significant differences for treatment indicated by *p < 0.05 and **p < 0.01. *Scale bar* 50 µm in panel **a**, which applies to all images

Given that structural blood vessel characteristics were impacted in offspring of mothers treated with dex during gestation (Fig. 4), it was important to assess the state of the BBB (Fig. 5). Importantly, the impact of fetal dex exposure on later BBB competency was opposite in the CTX versus PVN. In the CTX, there was statistically significant 12 % less extravascular FITC leakage in offspring from mothers treated with dex compared to those exposed to vehicle (Fig. 5a, d; *p* < 0.05). This suggests that there was an increase in the competency of the BBB due to dex-treatment in the CTX. In stark contrast, the mid region of the PVN showed a statistically significant 17 % increase in extravascular FITC leakage in dex-treated compared to vehicle-treated offspring (Fig. 5c, f, g; *p* < 0.05). There was a strong trend for prenatally dex-treated mice to have an increase in extravascular FITC in the rostral PVN compared to vehicle-treated (data not shown; *p* < 0.09) with no notable differences observed in the caudal PVN. For the LH, there was no change in extravascular FITC leakage in offspring from mothers either prenatally dex- or vehicle-treated (Fig. 5b, e). Due to the possibility of maternal injection providing a stressful stimulus that could increase endogenous glucocorticoid levels, a comparison was made between offspring of vehicle-injected mothers versus offspring from mothers who were not injected (Fig. 1). There were no differences in vascular characteristics or BBB competency when compared with non-injected mice. Together these findings suggest that fetal antecedent exposure to dex decreased the density and integrity of the blood vessels selectively within the PVN when examined in later life.

### Fetal dex exposure led to altered pericytes at P20

**Fig. 6:**
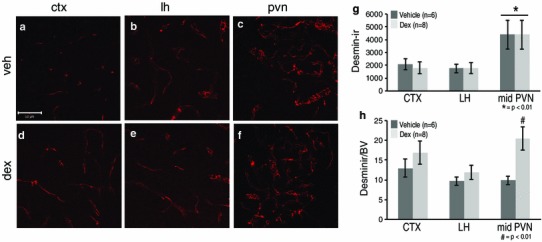
Prenatal exposure to dexamethasone (dex) impacted desmin-immunoreactive pericyte coverage in the mouse paraventricular nucleus of the hypothalamus (PVN) at P20. Example confocal images for each region are provided in panels **a**–**f**, and a quantitative summary by graph in **g** and **h**. In the PVN, there was a significant increase in desmin-immunoreactive pericyte coverage in dex-treated compared to vehicle-treated mice (**c**, **f**; *p < 0.01) when blood vessel density was taken into account (**h**; *p < 0.01). There were no significant differences observed in desmin-immunoreactive pericyte coverage in the cortex (CTX; **a**, **d**) or lateral hypothalamus (LH; **b**, **e**) between dex-treated or vehicle-treated mice. There was a significant increase in desmin-immunoreactive pericyte coverage in the PVN regardless of treatment compared to the CTX and LH (**g**). Number of animals per group is provided in the code for the *bars* in panels **g** and **h**. Significant differences between regions indicated by *asterisk* and for treatment as *hash*. *Scale bar* 50 µm in panel **a**, which applies to all images

To complement and further expand on the extravascular FITC data, desmin-immunoreactive pericyte coverage was assessed. Prenatal dex-treatment led to a significant increase in immunoreactive desmin on a vascular network that was less dense at P20 (Fig. 6). When total desmin immunoreactivity was examined in the PVN, LH or CTX, there were no dex-dependent differences in any region (Fig. 6a–g). However, when blood vessel density was taken into account, there was a significant dex-dependent increase in desmin-immunoreactive pericyte coverage in the mid PVN (Fig. 6h; *p* < 0.01). There were no significant differences in the rostral or caudal PVN due to treatment. There were also no significant differences in the CTX or LH due to treatment although there was a trend of increased coverage due to dex-treatment for all brain regions examined. Overall, prenatal dex-treated mice increased immunoreactive desmin on blood vessels within the PVN at P20.

## Discussion

Interest in the regulation of BBB function ranges from pharmaceutical perspectives for gaining or preventing drug access to the brain parenchyma (Abbott 2013), to questions of breakdown that might be antecedent to disorder (Gosselet et al. 2011; Daneman 2012; Abbott and Friedman 2012). The current study was focused on the PVN as a unique site that gains several fold greater vascular density than surrounding regions over the course of postnatal development. The increased vasculature might make changes in BBB function in this site particularly important. As the PVN may be particularly important as a site susceptible to fetal antecedent actions of excess glucocorticoids (Tobet et al. 2013), the current study also determined whether excess fetal glucocorticoids could impact PVN vascular characteristics. The results highlighted several critical points. First, that the development of vascular and BBB characteristics differed in the PVN versus the CTX. Secondly, that maternal exposure to excess glucocorticoids during pregnancy impacted vascular and BBB characteristics in their offspring. Thirdly, fetal exposure to dex impacted the CTX differentially than the PVN. Finally, alterations in BBB competency were paralleled by changes in pericyte coverage as assessed by immunoreactive desmin in development and as a function of fetal dex-treatment.

The ability of compounds to “leak” from blood vessels clearly differs among brain regions and can be observed using several methods. Differences in leakage among circumventricular organs were shown using FITC even though all are fenestrated and lack a BBB (Morita and Miyata 2012). Many BBB studies predominantly focus on the cortex for changes (e.g., Sadowska et al. 2009; Daneman et al. 2010; Vorbrodt et al. 2001; Ezan et al. 2012; Armulik et al. 2010; Bell et al. 2010) and occasionally examine the cerebellum (Sadowska et al. 2009; Armulik et al. 2010). For BBB development, reports indicate cortical leakage of high molecular weight dyes until postnatal day 21 in rats (Utsumi et al. 2000), and postnatal day 14 in mice (Lossinsky et al. 1986; Vorbrodt et al. 1986). The findings presented in this study also suggest that differences occur between brain regions such as the CTX and nuclear groups in the hypothalamus (i.e., LH and PVN). Not only were differences in BBB development observed, but fetal exposure to dex impacted the postnatal CTX differentially than the PVN and had little to no impact on the LH. This highlights the importance of studying cells in their anatomical context. For example, a number of studies have examined BBB competency by injecting Evans blue dye, perfusing saline to flush out circulating dye, and then homogenizing tissue to measure and analyze residual Evans blue in the tissue of interest (e.g., Bake and Sohrabji 2004). In the current study, this would have concealed differences between hypothalamic subregions. Overall, these findings suggest the need for further investigations to determine region-specific BBB development, how factors such as excess glucocorticoids during fetal development can impact BBB development, and what role this may play on an organism.

Prenatal glucocorticoid excess has been implicated in depression-like behaviors (Bale 2005), hypertension (Levitt et al. 1996), and hypothamalic-pituitary-adrenal axis dysregulation (Levy and Tasker 2012) in adulthood. Concerning the vasculature, previous work revealed decreased blood vessel density in the hippocampus (Neigh et al. 2010) and the germinal matrix at the level of the mid-septal nucleus (Vinukonda et al. 2010). In the current study, prenatal dex-treatment resulted in offspring for which the entire PVN had a reduced vascular network, albeit predominantly in the rostral and mid regions. Rostral and mid regions of the mouse PVN have a greater density of blood vessels and neurons that correspond with the general location of neuroendocrine neurons. By contrast, the caudal PVN is less densely vascular than rostral regions and houses more preautonomic neuronal populations important for sympathetic and parasympathetic outflow (Biag et al. 2012). The findings in the current study indicated that the blood vessel density and BBB within the caudal PVN were less impacted by excess glucocorticoids during development than rostral and mid regions. There were no changes observed in the LH or CTX. Even though prenatal dex-treatment is “global” in access, and impacts can be broad (physiology and behavior), the influences in the current study were selective within brain compartments.

The hallmark of capillaries is the ability to pass red blood cells in single file through tissues of the body. If red blood cells are the same size throughout, it is curious that not all capillaries in the brain have the same width. Nonetheless, the current results confirm a previous study in rats showing that capillaries of the PVN have larger lumens when compared with a region ventrolateral to the PVN (Van den Pol 1982). Although this may be due to a higher presence of venules (Ambach and Palkovits 1974), we did not make this determination. The results of the current study extended observations to mice, a comparison to CTX, and further showed that capillary widths were not altered due to prenatal dex-treatment even when total capillary volumes changed.

The developmental time course for BBB proteins varies for detectability and relationship to BBB competency. In previous studies in mice, BBB proteins did not reach adult levels in the CTX until around P14 (Vorbrodt et al. 2001). For the gap junction protein Connexin 30, immunoreactivity was detected in the mouse cortex beginning at postnatal P12 with the level of protein comparable to adulthood identified around P15 (Ezan et al. 2012). Results in the current study showed higher levels of extravascular FITC leakage occurring in the CTX at P12 than at P22, in agreement with the proposal that the BBB is still developing postnatally. At P52, compared to P12 and P22, the results showed that the BBB prevented FITC from entering the brain parenchyma in all regions examined. Prenatal exposure to dex impacted the BBB at P20 with less detectable extravascular FITC leakage in the CTX. In sheep CTX, prenatal dex resulted in an increase in tight junction proteins, a component of a functional BBB (Sadowska et al. 2009) and in agreement with the current findings. By contrast to the PVN, fetal dex led to the opposite result, greater extravascular FITC leakage suggesting BBB compromise. Insults such as excess prenatal glucocorticoid exposure can alter permeability and integrity in a brain region-dependent manner and for the PVN where the result is a less-dense vascular network that has a compromised BBB; the impact may alter physiology and behavior based on the neuronal population involved (Biag et al. 2012; Kádár et al. 2010; Tobet et al. 2013; Goldstein et al. 2013).

Prior studies examining pericytes found that fetal glucocorticoids increased cell coverage of NG2-positive pericytes in rabbits and humans (Vinukonda et al. 2010). While the prior report was in the germinal matrix for the cerebral cortex, the current study produced similar changes in the mid region of the PVN in mice. For pericytes, immunoreactive desmin suggests that changes have occurred, but not whether the number, distribution, or size of pericytes was impacted. One explanation for why there is the same level of desmin immunoreactivity in the PVN on fewer blood vessels due to prenatal dex-treatment may be due to recruitment and migration. Pericytes migrate in response to new vessel formation, traumatic stress, or under hypoxic injury or state (Dore-Duffy et al. 2000). In dex-treated offspring that exhibited a decreased vascular network, this may be a sign of prior hypoxia with pericyte recruitment needed to promote recovery. Enhanced pericyte coverage may serve to help stabilize the vasculature (Vinukonda et al. 2010). Since pericytes can regulate capillary diameter through constricting the vascular wall (Bell et al. 2010), differences due to prenatal glucocorticoid excess may impact blood flow within the PVN. Future studies are needed to determine how changes in desmin-positive pericyte coverage in dex-treated offspring impacts the ability of the BBB to function properly as observed here through extravascular FITC leakage and whether this impacts neuronal function.

In summary, the current study examined the postnatal development of the BBB and demonstrated that fetal dex exposure altered the integrity of the BBB in the PVN. There was an increase in BBB permeability at P20 in the highly vascularized middle region of the PVN. Decreases in blood vessel density and BBB integrity within the mid (and to some extent rostral) regions of the PVN may impact the ability of neuroendocrine neurons (Biag et al. 2012; Kádár et al. 2010) to function normally. Understanding changes in the crosstalk between neurons and blood vessels in the PVN may provide insight into the long-term behavioral and physiological consequences observed in human and animal studies when exposed to glucocorticoid excess during prenatal development.

## Electronic supplementary material

Below is the link to the electronic supplementary material.

Supplemental Fig. 1 Regions selected for analysis. Brightfield image of a coronal section immunolabeled for Platelet Endothelial Cell Adhesion Molecule to visualize blood vessels. Boxes show representative regions of the Cortex (CTX), Lateral Hypothalamus (LH), and Paraventricular Nucleus of the Hypothalamus (PVN) that were selected for analysis.

Supplemental Fig. 2. Analysis of vascular permeability. Fluorescence intensities were measured outside of blood vessels (i.e., leak) in the Cortex (CTX), Lateral Hypothalamus (LH) and Paraventricular Nucleus of the Hypothalamus (PVN). Semi-automated leakage calculations were made using CellProfiler software. Blood vessels were identified in panels a-c, a 10-pixel expansion was mapped from each blood vessel to create a mask and intensity was measured as shown in panel d, and blood vessel intensity was then subtracted as in panel e to generate blood–brain barrier permeability quantification.

Supplementary material 1 (PDF 899 kb)

Supplementary material 2 (PDF 4841 kb)
