# Supplementary figures and images for: Development of the blood–brain barrier within the paraventricular nucleus of the hypothalamus: influence of fetal glucocorticoid excess
Source: Brain Struct Funct. 2014 May 11;220(4):2225–34. doi: 10.1007/s00429-014-0787-8 (PMC4481307; doi:10.1007/s00429-014-0787-8)

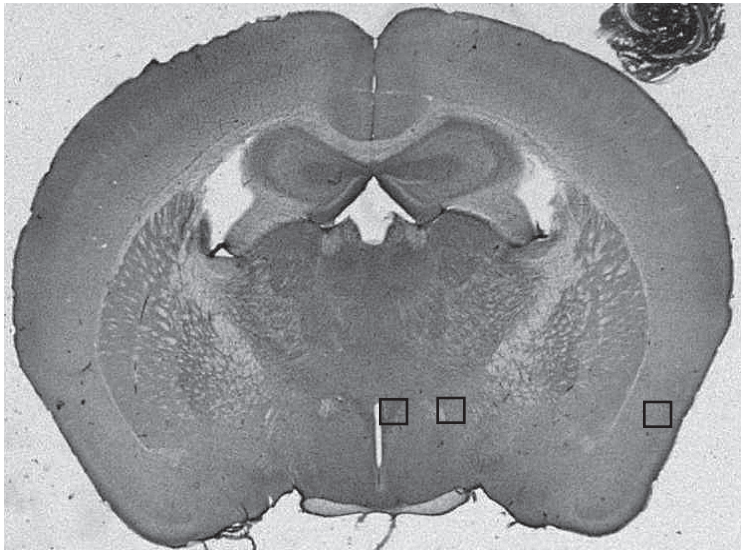

Supplement: Supplementary file 1 — Supplementary material 1 (PDF 899 kb) [file 429_2014_787_MOESM1_ESM.pdf]

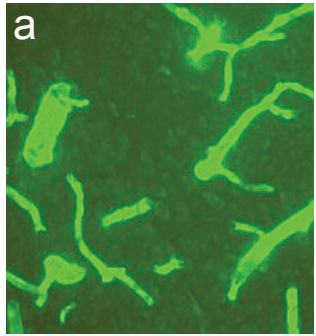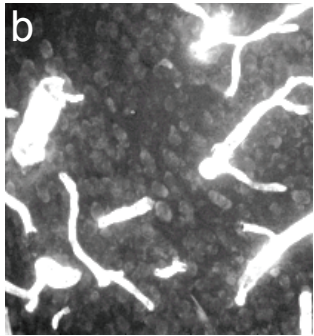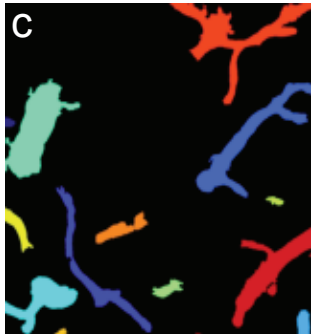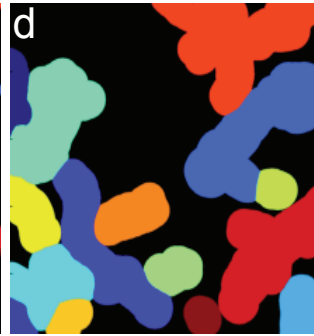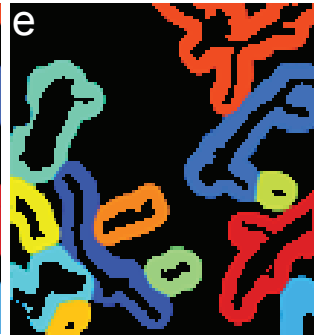

Supplement: Supplementary file 2 — Supplementary material 2 (PDF 4841 kb) [file 429_2014_787_MOESM2_ESM.pdf]
